# Early or late initiation of dabigatran versus vitamin-K-antagonists in acute ischemic stroke or TIA: The PRODAST study

**DOI:** 10.1177/17474930231184366

**Published:** 2023-07-04

**Authors:** Gerrit M Grosse, Anika Hüsing, Andreas Stang, Nils Kuklik, Marcus Brinkmann, Darius Nabavi, Paul Sparenberg, Karin Weissenborn, Klaus Gröschel, Georg Royl, Sven Poli, Dominik Michalski, Christoph C Eschenfelder, Christian Weimar, Hans-Christoph Diener, on behalf of the PRODAST Investigators

**Affiliations:** 1 Department of Neuroepidemiology, Institute for Medical Informatics, Biometry and Epidemiology, Faculty of Medicine, University of Duisburg-Essen, Essen, Germany; 2 Department of Neurology, Hannover Medical School, Hannover, Germany; 3 Department of Epidemiology, School of Public Health, Boston University, Boston, MA, USA; 4 Center for Clinical Trials Essen, University Hospital Essen, Essen, Germany; 5 Department of Neurology, Vivantes Klinikum Neukölln, Berlin, Germany; 6 Department of Neurology, BG Klinikum Unfallkrankenhaus Berlin, Berlin, Germany; 7 Department of Neurology, University Medical Center of the Johannes Gutenberg University Mainz, Mainz, Germany; 8 Department of Neurology, University of Lübeck, Lübeck, Germany; 9 Department of Neurology and Stroke, University of Tübingen, Tübingen, Germany; 10 Hertie Institute for Clinical Brain Research, University of Tübingen, Tübingen, Germany; 11 Department of Neurology, University of Leipzig, Leipzig, Germany; 12 Human Pharma Germany, Boehringer Ingelheim Pharma GmbH & Co. KG, Ingelheim, Germany; 13 BDH-Klinik Elzach, Elzach, Germany

**Keywords:** Acute ischemic stroke, anticoagulation, dabigatran, hemorrhagic transformation, secondary prevention, intracranial hemorrhage, transient ischemic attack, vitamin K antagonists

## Abstract

**Background:**

The optimal timing of initiating or resuming anticoagulation after acute ischemic stroke (AIS) or transient ischemic attack (TIA) in patients with atrial fibrillation (AF) is debated. Dabigatran, a non-vitamin K oral anticoagulant (NOAC), has shown superiority against vitamin K antagonists (VKA) regarding hemorrhagic complications.

**Aims:**

In this registry study, we investigated the initiation of dabigatran in the early phase after AIS or TIA.

**Methods:**

PRODAST (Prospective Record of the Use of Dabigatran in Patients with Acute Stroke or TIA) is a prospective, multicenter, observational, post-authorization safety study. We recruited 10,039 patients at 86 German stroke units between July 2015 and November 2020. A total of 3,312 patients were treated with dabigatran or VKA and were eligible for the analysis that investigates risks for major hemorrhagic events within 3 months after early (⩽ 7 days) or late (> 7 days) initiation of dabigatran or VKA initiated at any time. Further endpoints were recurrent stroke, ischemic stroke, TIA, systemic embolism, myocardial infarction, death, and a composite endpoint of stroke, systemic embolism, life-threatening bleeding and death.

**Results:**

Major bleeding event rates per 10,000 treatment days ranged from 1.9 for late administered dabigatran to 4.9 for VKA. Early or late initiation of dabigatran was associated with a lower hazard for major hemorrhages as compared to VKA use. The difference was pronounced for intracranial hemorrhages with an adjusted hazard ratio (HR) of 0.47 (95% confidence interval (CI): 0.10–2.21) for early dabigatran use versus VKA use and an adjusted HR of 0.09 (95% CI: 0.00–13.11) for late dabigatran use versus VKA use. No differences were found between early initiation of dabigatran versus VKA use regarding ischemic endpoints.

**Conclusions:**

The early application of dabigatran appears to be safer than VKA administered at any time point with regards to the risk of hemorrhagic complications and in particular for intracranial hemorrhage. This result, however, must be interpreted with caution in view of the low precision of the estimate.

## Introduction

The timing of initiating or resuming of anticoagulation after an acute ischemic stroke (AIS) or transient ischemic attack (TIA) in patients with atrial fibrillation (AF) is still a matter of debate. Early initiation of anticoagulation may increase the risk of hemorrhagic transformation (HT) of the infarct and thus intracranial hemorrhage (ICH). Delaying the initiation of anticoagulation, however, may enhance the risk of recurrent ischemic events.^1^ With one exception,^2^ results from randomized controlled trials (RCTs) on the timing of anticoagulation^3–5^ are not yet available and guidelines are currently based on expert opinion.^6–8^ Surveys among stroke practitioners on timing of anticoagulation following AIS or TIA have shown no consensus in decision-making.^9,10^

Observational studies on the initiation of anticoagulation after AIS or TIA in patients with AF yielded heterogeneous findings^1,11–14^ and the majority of these studies were conducted in the era before non-vitamin K dependent anticoagulants (NOAC) replaced vitamin K antagonists (VKA). NOAC are safer compared to VKA with regard to major hemorrhagic complications.^15,16^ However, the pivotal trials leading to the approval of NOAC in patients with AF excluded patients with AIS occurring 7–14 days before initiation of treatment.^17–20^ Importantly, real-world data on the application of NOAC in the first week after AIS or TIA are scarce. We hypothesized that the hazards for major hemorrhages, most importantly ICH, may also be substantially lower in patients in whom NOAC are initiated early after AIS or TIA compared to patients who receive VKA.

## Aims

The Prospective Record of the Use of Dabigatran in Patients with Acute Stroke or TIA (PRODAST; ClinicalTrials.gov Identifier: NCT02507856) study was initiated to provide real-world evidence on this issue. The main objective of PRODAST was the comparison of the 3-month rates of major hemorrhagic events between an early (⩽ 7 days) or late (> 7 days) initiation of dabigatran in comparison to treatment with VKA started at any time, in patients with AF and a recent AIS or TIA (⩽ 7 days). In addition to this primary objective, we investigated additional endpoints, such as ischemic stroke, TIA, systemic embolism, myocardial infarction, and HT, as well as the survival status depending on treatment. We aimed to provide real-world evidence on the early treatment with anticoagulants in this vulnerable phase of AIS and TIA.

## Methods

### Study design

Methods and the study design have been published in detail.^21^ PRODAST is a multi-center prospective, observational, non-interventional post-authorization safety study (PASS), which recruited patients with AF who experienced a recent (⩽ 7 days) AIS or TIA with AF at 86 German stroke units between July 2015 and November 2020. Inclusion criteria were age ⩾ 18 years at enrollment, written informed consent (IC), AIS or TIA within 7 days before enrollment and a diagnosis of non-valvular AF. Exclusion criteria included the presence of mechanical heart valves or valve disease that was expected to require valve replacement intervention (surgical or non-surgical) during the next 3 months. Patients who were participating in any RCT of an experimental drug or device, women of childbearing age without anamnestic exclusion of pregnancy, or who were not using an effective contraception, as well as nursing mothers, were also excluded. All therapeutic procedures were at the discretion of the treating physicians. Documentation of effective anticoagulation was also the responsibility of the respective study centers. For all patients, detailed demographic and clinical data were collected during their hospital stay, as previously described in detail.^21^ All patients except those discharged with an NOAC other than dabigatran underwent a standardized follow-up after 3 months as well as an assessment of vital status after one year, according to the scope of the PASS design. For the analysis of the primary study objective, all patients who were treated with dabigatran or VKA were considered and outcomes of patients on NOAC other than dabigatran, that is, factor Xa inhibitors, will be reported elsewhere.

### Outcomes

The primary endpoint was defined as major bleeding event within 3 months following the index event. Major bleeding events were defined as any of the following: a fatal bleeding; intracranial, intraocular, intraspinal, retroperitoneal, intraarticular or intramuscular bleeding causing a compartment syndrome; or clinically overt bleeding associated with either a decrease in hemoglobin concentration of > 2 g/dL, indication for transfusion of two or more units of whole blood or packed cells, or indication for surgical intervention. Secondary endpoints were recurrent strokes, recurrent ischemic strokes, TIA, systemic/pulmonary embolism, myocardial infarction, death, and the composite endpoint, which consisted of stroke, systemic embolism, life-threatening bleeding and death. Endpoints occurring during hospitalization were assessed by the respective study sites. Following central and on-site data verification, unclear outcome events were confirmed by an independent clinical adjudication committee, taking into account all available information. In addition, in unclear cases, medical records were retrieved from the general practitioner.

### Ethical approval

All patients or their legal representatives provided written IC. In the case that IC could not be obtained in a timely manner due to the patient’s condition, the appropriate investigator was allowed to decide on study inclusion, whereupon the IC process was rescheduled as soon as possible. Ethical approval was provided by the institutional review board of the University of Duisburg-Essen (No. 15-6202-BO). All procedures were conducted in accordance with the national law, the 1964 Declaration of Helsinki and its later amendments, and the recommendations of the guidelines on Good Clinical Practice and Good Epidemiological Practice.

### Statistical analysis

Two types of analyses were performed: First, we analyzed the initiation-time-specific effects of dabigatran therapy in comparison to VKA treatment. For this, a distinction was made between dabigatran treatment initiated early, that is, ⩽ 7 days, and late, that is, > 7 days, and exposure was statistically considered as persistent from the first day of dabigatran therapy until the end of the patient’s follow-up. Prevalent use of dabigatran at the time of the index event was considered as early dabigatran application. Second, we performed an analysis directly comparing events under actual (current) treatment with dabigatran with VKA independent of the initiation time. Each patient’s observed time under the respective treatment regimen was considered starting at the index event, from the day of treatment initiation, until the occurrence of the endpoint event, censoring due to treatment change, an alternative severe event (i.e. any endpoint, except TIA), or end of follow-up. Hazard ratios (HR) were estimated using crude and adjusted Cox-proportional hazards regression analyses. Crude models were adjusted for age, study site (as random effect), and time-varying antithrombotic medication in the categories NOAC, VKA, antiplatelets, and non-oral antithrombotic medication. Adjusted models included additional risk factors to correct for confounding or treatment by indication bias based on directed acyclic graphs (DAGs) as described previously^21^ (see online Supplemental Figure 1 and adjustment matrix Supplemental Table 1). To account for the varying duration of the effect of antithrombotic drugs beyond intake, the respective end of therapy was postponed by a lag time, as previously described^21^ (see online Supplemental Table 2).

All analyses were conducted using SAS 9.4 (SAS Institute Inc., Cary, NC, USA).

## Results

### Patients’ characteristics

We recruited 10,039 patients in the PRODAST study. A total of 3,312 patients were treated with dabigatran or VKA and were thus eligible for the analysis of the primary study objective. Analyses regarding administration of other anticoagulants will be published separately. Overall, 459 patient-years of dabigatran users and 337 patient-years of VKA users were analyzed. Demographical and clinical characteristics of the study population are shown in Table 1. Patients with late initiation of dabigatran more frequently underwent mechanical thrombectomy or thrombolysis, or had early HT shown by initial cranial imaging. Infarct volumes and the National Institutes of Health Stroke Scale (NIHSS) scores were higher in patients who received dabigatran late (median NIHSS score: 5) as compared to patients who received dabigatran early or VKA (median NIHSS score: 2 and 2, respectively). These observations are suggestive of confounding by indication. Supplemental Table 3 provides an overview on the actual treatment times of different anticoagulants according to the patient groups.

**Table 1.** Patients’ demographic and clinical characteristics of the PRODAST study, Germany, 2015–2022.

	Dabigatran (early)	Dabigatran (late)	VKA
*N*	1642	274	1396
Age (years, median (5th–95th percentile))	76 (55–89)	76 (56–89)	80 (63–90)
Sex (female (%))	700 (43%)	135 (49%)	599 (43%)
Arterial hypertension	1317 (80%)	226 (82%)	1248 (89%)
Diabetes mellitus	408 (25%)	59 (22%)	517 (37%)
Congestive heart failure	145 (9%)	25 (9%)	244 (17%)
Renal insufficiency	12 (1%)	4 (1%)	131 (9%)
Liver insufficiency	16 (1%)	2 (1%)	12 (1%)
Dyslipidemia	686 (42%)	101 (37%)	587 (42%)
Current smoker	168 (10%)	25 (10%)	99 (7%)
Previous smoker	463 (28%)	78 (28%)	413 (30%)
BMI (kg/m², median (5th–95th percentile))	26.70 (20.90–36.30)	27.30 (21.00–37.70)	26.50 (20.90–36.50)
History of ischemic stroke	320 (19%)	44 (16%)	332 (24%)
History of hemorrhagic stroke	11 (1%)	1 (0%)	5 (0%)
Potential symptomatic arterial stenosis	189 (12%)	38 (14%)	185 (13%)
TIA	409 (25%)	10 (4%)	396 (28%)
National Institutes of Health Stroke Scale (NIHSS) (median (5th–95th percentile))	2 (0–15) (2.1% missing)	5 (0–18) (2.2% missing)	2 (0–16) (1.6% missing)
Infarct volume (mL; median (5th–95th percentile))	0.420 (0.008–12.000) (57.7% missing)	2.940 (0.030–36.000) (28.8% missing)	0.675 (0.008–36.800) (62.0% missing)
Early hemorrhagic transformation (HT)	63 (4%)	42 (15%)	74 (5%)
Thrombolysis	344 (21%)	87 (32%)	110 (8%)
Mechanical thrombectomy	194 (12%)	68 (25%)	124 (9%)
CHA_2_DS_2_VASc score (median (5th–95th percentile))	5 (3–7)	5 (3–7)	6 (4–7)
Prevalent use of ATT	720 (44%)	72 (26%)	1220 (87%)
Observed patient time (days, median (5th–95th percentile) (sum))	92 (6–96) (143,128)	92 (16–100) (24,470)	92 (4–211) (122,954)
Days from index event until discharge from hospital (days, median (5th–95th percentile) (sum))	6 (2–17) (11,946)	11 (5–30) (3636)	7 (2–20) (11,799)
Days from index event until initiation of ATT (days, median (5th–95th percentile))	3 (0–7)	10 (8–30)	5 (1–16)

All data are *n* (%) unless specified.

PRODAST: Prospective Record of the Use of Dabigatran in Patients with Acute Stroke or TIA; VKA: vitamin K antagonists; BMI: body mass index; TIA: transient ischemic attack; NIHSS: National Institutes of Health Stroke Scale; HT: hemorrhagic transformation; CHA_2_DS_2_VASc: congestive heart failure, hypertension, age ⩾ 75 (doubled), diabetes, stroke (doubled), vascular disease, age 65 to 74 and sex category (female); ATT: antithrombotic therapy.

### Incidence rates of endpoint events after treatment initiation or resumption

As shown in Table 2, the crude incidence rates of endpoint events were very low. We observed only three major bleeding events after late initiation of dabigatran, resulting in rates per 10,000 treatment days from 1.9 for late administered dabigatran to 4.9 for VKA (26 events in total). Secondary endpoints were also infrequent, with 7 and 42 stroke recurrences after late and early initiation of dabigatran treatment, respectively, resulting in event rates of 3.8 after early dabigatran initiation, 4.4 after late dabigatran initiation, and 5.8 per 10,000 treatment days under treatment with VKA. Ischemic stroke rates were lower: 3.3, 4.4 and 4.5 per 10,000 treatment days after early dabigatran initiation, late dabigatran initiation and VKA treatment, respectively. After late initiation of dabigatran, no TIAs were observed and there was only one case each of systemic embolism and myocardial infarction. Thirty-day mortality rates were 3.4 per 10,000 treatment days with VKA, compared with 1.9 and 2.2 after early and late initiation of dabigatran, respectively.

**Table 2.** Incidence rates of endpoint events after treatment initiation (events/10,000 treatment days), PRODAST Study, Germany, 2015–2020.

	Dabigatran (early)	Dabigatran (late)	Dabigatran (current use)	VKA (current use)
Cumulative treatment days	110,736	15,958	113,530	53,312
Major hemorrhage (cases)	2.2 (24)	1.9 (3)	2.0 (23)	4.9 (26)
ICH (cases)	0.5 (6)	0.6 (1)	0.4 (5)	1.9 (10)
Hemorrhagic transformation (cases)	0.3 (3)	0 (0)	0.3 (3)	0.9 (5)
Life-threatening bleeding (cases)	0.7 (8)	1.3 (2)	0.6 (7)	2.8 (15)
Gastrointestinal bleeding (cases)	1.2 (13)	1.3 (2)	1.2 (14)	1.5 (8)
Stroke (cases)	3.8 (42)	4.4 (7)	3.7 (42)	5.8 (31)
Ischemic stroke (cases)	3.3 (37)	4.4 (7)	3.4 (39)	4.5 (24)
TIA (cases)	1.5 (17)	0 (0)	1.3 (15)	2.8 (15)
Myocardial infarction (cases)	0.8 (9)	0.6 (1)	0.8 (9)	1.5 (8)
Systemic embolism (cases)	0.5 (6)	0.6 (1)	0.4 (5)	0.4 (2)
Mortality (cases)	1.9 (22)	2.2 (4)	1.4 (17)	3.4 (19)
Composite endpoint (cases)	8.5 (95)	7.5 (12)	8.0 (78)	14.5 (78)

PRODAST: Prospective Record of the Use of Dabigatran in Patients with Acute Stroke or TIA; VKA: vitamin K anticoagulant; ICH: intracranial hemorrhage; TIA: transient ischemic attack.

### Comparison of early and late initiation of dabigatran with VKA use

**Figure 1.**
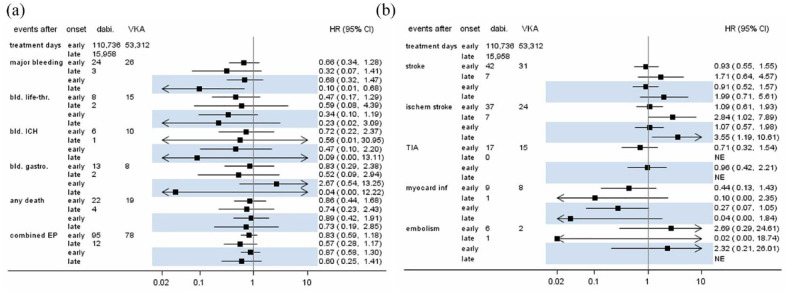
Case numbers and estimated hazard ratios (with 95% confidence intervals) for early/late use of dabigatran compared to current use of VKA. Highlighted estimates are from adjusted models. (a) Hemorrhagic endpoints, death, and composite endpoint; and (b) ischemic endpoints. CI: confidence interval; EP: endpoint; HR: hazard ratio; ICH: intracranial hemorrhage; NE: not estimable; TIA: transient ischemic attack; VKA: vitamin K antagonists.

Figure 1 presents crude and adjusted effect sizes for early and late initiation of dabigatran versus VKA use. Early or late initiation of dabigatran was associated with a lower rate of major hemorrhages compared with VKA use, according to the crude and adjusted analyses (early dabigatran initiation, crude HR (cHR) 0.66; 95% confidence interval (CI): 0.34–1.28 and adjusted HR (aHR) 0.68; 95% CI: 0.32–1.47; late dabigatran initiation, cHR: 0.32; 95% CI: 0.07–1.41 and aHR: 0.10; 95% CI: 0.01–0.68). This effect was even more pronounced for the endpoint of ICH with an aHR of 0.47 (95% CI: 0.10–2.21) for early dabigatran use versus VKA use and an aHR of 0.09 (95% CI: 0.00–13.11) for late dabigatran initiation versus VKA use. Early initiation of dabigatran was associated with a lower rate of HT (aHR: 0.51; 95% CI: 0.03–7.96) compared with VKA. No HT occurred following late initiation of dabigatran. The effect estimates for systemic embolism were imprecise due to the low number of events. No differences between early initiation of dabigatran versus VKA use were found regarding stroke (aHR: 0.91; 95% CI: 0.52–1.57), ischemic stroke (aHR: 1.07; 95% CI: 0.57–1.98) or TIA (aHR: 0.96; 95% CI: 0.42–2.21). Late initiation of dabigatran resulted in a higher rate of ischemic strokes compared with VKA use (aHR: 3.55; 95% CI: 1.19–10.61). Both early and late dabigatran initiation were associated with lower rates of myocardial infarction compared with VKA treatment (early initiation aHR: 0.27; 95% CI: 0.07–1.05; late initiation aHR: 0.04; 95% CI: 0.00–1.84). The rates of death and composite endpoints were reduced after late initiation of dabigatran compared with VKA use (death aHR: 0.73; 95% CI: 0.19–2.85; composite endpoint aHR: 0.60; 95% CI: 0.25–1.41).

### Comparison between current use of dabigatran and VKA

**Figure 2.**
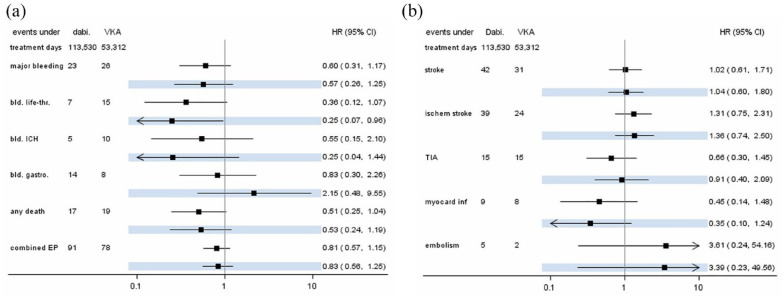
Case numbers and hazard ratio estimates (with 95% confidence intervals) for current use of dabigatran compared to current use of VKA. Highlighted estimates are from adjusted models. (a) Hemorrhagic endpoints, death, and composite endpoint; and (b) ischemic endpoints. CI: confidence interval; EP: endpoint; HR: hazard ratio; ICH: intracranial hemorrhage; TIA: transient ischemic attack; VKA: vitamin K antagonists.

Figure 2 presents crude and adjusted effect estimates for current use of dabigatran versus VKA. The rate of major hemorrhage was reduced by 43% when comparing current use of dabigatran with VKA (aHR: 0.57; 95% CI 0.26–1.25). This effect was even more pronounced for the endpoint ICH, which had a rate reduction of 75% (aHR: 0.25; 95% CI: 0.04–1.44). The rate of HT was also substantially reduced by dabigatran compared with VKA (aHR: 0.25; 95% CI: 0.02–2.99). The rates of AIS and TIA were not different between current dabigatran use (AIS aHR: 1.36; 95% CI: 0.74–2.50; TIA aHR: 0.91; 95% CI: 0.40–2.09) and VKA use. Dabigatran use had a preventive effect on incident myocardial infarctions compared with VKA (aHR: 0.35; 95% CI: 0.10–1.24), whereas the effect estimation was imprecise for incident systemic embolisms (aHR: 3.39; 95% CI: 0.23–49.56) due to small number of events. The mortality rate was nearly halved by use of dabigatran compared with VKA (aHR: 0.53; 95% CI: 0.24–1.19) and the composite endpoint occurred less frequently (aHR: 0.83; 95% CI: 0.56–1.25).

## Discussion

The main findings of this study are: (1) In the real-world setting, rates of recurrent vascular events are generally low following initiation of anticoagulants in patients with AIS or TIA due to AF, (2) irrespective of the timing of initiation, dabigatran appears to be associated with a lower hazard for major hemorrhages as compared to VKA use, (3) this is especially true for intracranial hemorrhages, (4) there are no substantial differences between early initiation of dabigatran versus VKA use regarding ischemic endpoints. Anticoagulation with NOAC in patients with AF has been shown superiority against the use of VKA concerning major hemorrhagic complications.^15^ However, prior to this study, there was still uncertainty, whether the initiation of anticoagulation with NOAC is safe also in the very early phase after AIS or TIA. In the RCTs that led to the approval of NOAC, patients with recent AIS were excluded. Large observational studies, which focused on the use of NOAC, are limited and also included almost exclusively patients in whom the stroke had happened weeks before.^22^ The recently published *Timing of Oral Anticoagulant Therapy in Acute Ischemic Stroke With Atrial Fibrillation* (*TIMING*) study was the first RCT on this topic and showed that early initiation of anticoagulation was noninferior to delayed initiation concerning a composite endpoint of ischemic stroke, death, and ICH.^2^ The results of other currently ongoing RCTs, that is, ELAN,^5^ OPTIMAS,^4^ and START,^3^ are eagerly awaited. Real-world evidence reflecting the initiation or resumption of anticoagulants in daily practice is crucial as a complement to data derived from RCTs. Precisely, in this study, we were interested in whether an early start of dabigatran after AIS or TIA is safer than use of VKA.

PRODAST is the largest prospective study, which is dedicated to the issue of anticoagulation in the early phase following AIS or TIA in patients with AF. In this analysis focusing on the initiation of dabigatran in comparison to VKA, we were able to investigate data from in total 3312 patients for whom detailed characteristics and follow-up data for up to 3 months were prospectively collected.

Our data show that, in general, the rates of recurrent vascular events were very low following initiation of anticoagulants. Compared to previous observational studies which were conducted in the pre-NOAC era,^1,11^ the rates of outcome events in PRODAST are substantially lower, which must be interpreted as a recent improvement of secondary stroke prevention in routine clinical practice.

In the Randomized Evaluation of Long Term Anticoagulant Therapy With Dabigatran etexilate (RE-LY) trial that led to the approval of dabigatran for secondary stroke prevention, dabigatran was at least as effective in preventing AIS and safer in regard to hemorrhagic complications compared to warfarin.^17^ Our analysis now provides evidence that this observation holds true also in the early phase following AIS and TIA in a real-world setting. This is in accordance with findings from a pooled analysis of different observational studies.^23^ One shortcoming of observational studies is confounding by indication. This becomes apparent in the patient characteristics, which show that physicians favored later use of dabigatran in patients with more severe strokes. Patients who were treated with thrombolysis or mechanical thrombectomy, and those who already had HT shown by initial imaging, were more likely to receive anticoagulation later. To account for the complex causal structures behind this decision-making, we applied DAGs in detail to identify appropriate adjustment sets to estimate the total effect of the use of anticoagulation on the endpoints.

When comparing early or late initiation of dabigatran with VKA use, the rates for major hemorrhagic events were substantially lower in dabigatran-treated patients after adjustment for confounders. The rate for ICH was reduced by 54%, and 91% when comparing early or late dabigatran initiation with VKA use, respectively. The observation that delayed initiation with dabigatran may be associated with higher rates of ischemic stroke is consistent with clinical experience and existing evidence from previous observational studies.^1^

Considering outcome events occurring under the current treatments, we observed similar results: compared with VKA, current use of dabigatran reduced the rate of major hemorrhages, in particular ICH, myocardial infarction, and death. Ischemic strokes, however, were not prevented by current dabigatran treatment compared with VKA treatment.

The clear strength of our study is the multicenter, prospective design, which accurately represents the real-world administration of anticoagulants after AIS and TIA in a large sample. Nevertheless, despite the large sample size, the precision of estimates was low for all endpoint analyses due to the low number of events. Therefore, the findings must be interpreted with caution and require replication in analyses of larger samples. Of note, the average severity of stroke was relatively low in all groups, constraining the generalizability of the results to more severely affected patients. Further limitations arise from the observational design, which may lead to potential residual confounding despite all efforts to address these in multivariable models and the application of DAGs. The detailed collection of covariates allowed a stringent adjustment for de-confounding.

In conclusion, in this large prospective real-world study, the rates of recurrent vascular events were generally low after acute AIS or TIA in patients with AF who were treated with dabigatran or VKA. After adjustment for confounders, the early initiation of dabigatran appeared to be safer than VKA administered at any time point with regards to hemorrhagic complications, including ICH. However, due to the low precision of the estimation, this result must be interpreted with caution. In accordance with the results of the RE-LY study, incidences of ischemic endpoints were not different between dabigatran and VKA use. Additional analyses of the PRODAST study, as well as the results of the currently awaited RCTs, will provide further evidence on NOAC initiation after cardio-embolic AIS and TIA. More precisely, we will investigate the ideal starting time to initiate NOAC treatment following AIS or TIA, as a complement to the TIMING study and the other currently awaited RCTs.

## Supplemental Material

sj-docx-1-wso-10.1177_17474930231184366 – Supplemental material for Early or late initiation of dabigatran versus vitamin-K-antagonists in acute ischemic stroke or TIA: The PRODAST study Click here for additional data file. Supplemental material, sj-docx-1-wso-10.1177_17474930231184366 for Early or late initiation of dabigatran versus vitamin-K-antagonists in acute ischemic stroke or TIA: The PRODAST study by Gerrit M Grosse, Anika Hüsing, Andreas Stang, Nils Kuklik, Marcus Brinkmann, Darius Nabavi, Paul Sparenberg, Karin Weissenborn, Klaus Gröschel, Georg Royl, Sven Poli, Dominik Michalski, Christoph C Eschenfelder, Christian Weimar and Hans-Christoph Diener in International Journal of Stroke
