# Supplementary material for: Early or late initiation of dabigatran versus vitamin-K-antagonists in acute ischemic stroke or TIA: The PRODAST study
Source: Int J Stroke. 2023 Jul 4;18(10):1169–77. doi: 10.1177/17474930231184366 (PMC10676026; doi:10.1177/17474930231184366)
Supplement: sj-docx-1-wso-10.1177_17474930231184366 – Supplemental material for Early or late initiation of dabigatran versus vitamin-K-antagonists in acute ischemic stroke or TIA: The PRODAST study [file sj-docx-1-wso-10.1177_17474930231184366.docx]

**Online supplement**

**Supplemental table 1: Adjustment matrix for endpoint analyses as obtained by directed acyclic graphs.**


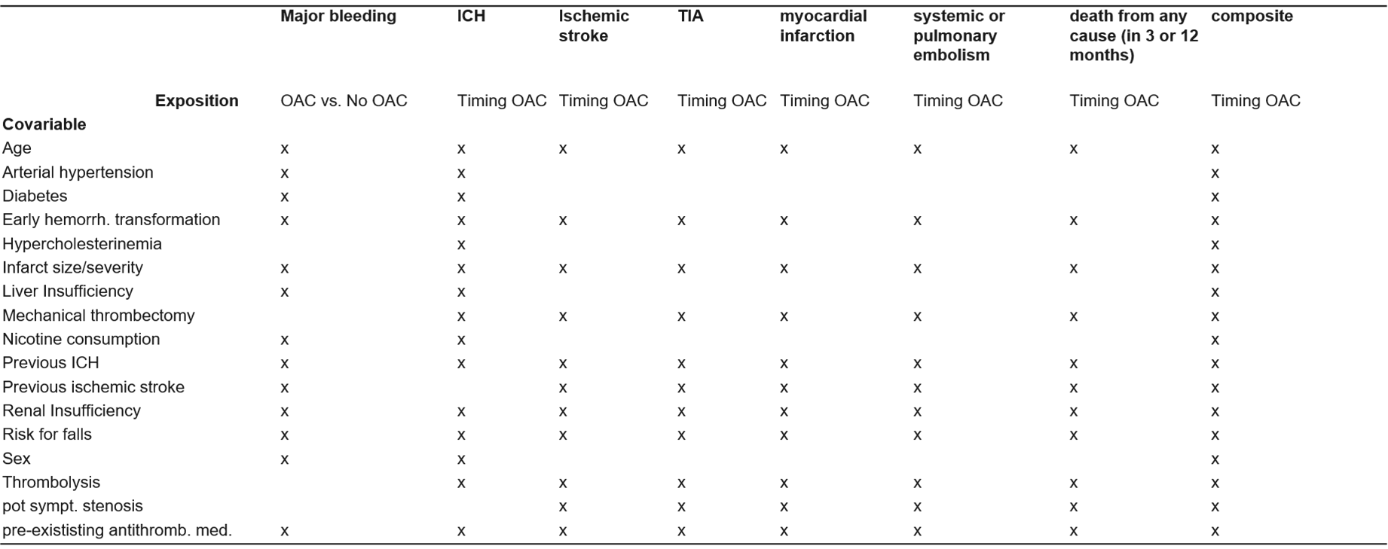


**Supplemental table 2: Delayed antithrombotic treatment effects after single dose or long-term therapy.** Replicated from Grosse et al. ^21^ under the terms of the creative commons licence.

|  | **Single dose** | **Long-term therapy** |
| --- | --- | --- |
| Vitamin-K oral anticoagulants | 0d | 5d  (after continuous treatment >3d) |
| Dabigatran | 1d | 1d |
| Other non-vitamin-K oral anticoagulants | 1d | 1d |
| Unfractionated Heparin | <1d | <1d |
| Low molecular weight Heparin | 1d | 1d |
| Antiplatelet therapy | 7d | 7d |

**Supplemental table 3: Duration of distinct antithrombotic treatments**

|  |  | **Dabigatran early** | **Dabigatran late** | **VKA** |
| --- | --- | --- | --- | --- |
| Time from index event to hospital discharge in days | median (5th- 95th percentile) | 6.0 (2.0 - 17.0) | 11.0 (5.0 - 30.0) | 7.0 (2.0 - 20.0) |
| Days to first ATT administration during hospitalization with reference to main groups, index = day 0 | median (5th- 95th percentile) | 3.0 (0.0 - 7.0) | 10.0 (8.0 - 30.0) | 5.0 (1.0 - 16.0) |
| **Dabigatran** | N | 1642 | 274 | 0 |
| Total duration of dabigatran use in days | median (5th- 95th percentile) | 87.0 (2.0 - 92.0) | 80.0 (2.0 - 84.0) | N/A |
| **VKA** | N | 195 | 41 | 1395 |
| Total duration of VKA use in days | median (5th- 95th percentile) | 2.0 (1.0 - 86.0) | 6.0 (1.0 - 82.0) | 7.0 (1.0 - 92.0) |
| **FX10 inhibitor** | N | 461 | 67 | 604 |
| Total duration of use of other FX10 inhibitors in days | median (5th- 95th percentile) | 5.0 (1.0 - 89.0) | 23.0 (1.0 - 84.0) | 5.0 (1.0 - 88.0) |
| **Oral antiplatelets** | N | 729 | 181 | 394 |
| Total duration of use of oral antiplatelets in days | median (5th- 95th percentile) | 4.0 (1.0 - 91.0) | 9.0 (2.0 - 91.0) | 8.0 (1.0 - 92.0) |
| **Non-oral antithrombotic therapy** | N | 765 | 203 | 593 |
| Total duration of non-oral antithrombotic therapy in days | median (5th- 95th percentile) | 3.0 (1.0 - 13.0) | 9.0 (3.0 - 36.0) | 7.0 (1.0 - 84.0) |

**Supplemental figure 1:** Directed acyclic graph (DAG) for identifying the minimal sufficient adjustment set for estimating the total effect of timing of anticoagulation on the occurrence of major bleeding events. Created using: http://www.dagitty.net/. Replicated from Grosse et al. ^21^ under the terms of the creative commons licence.


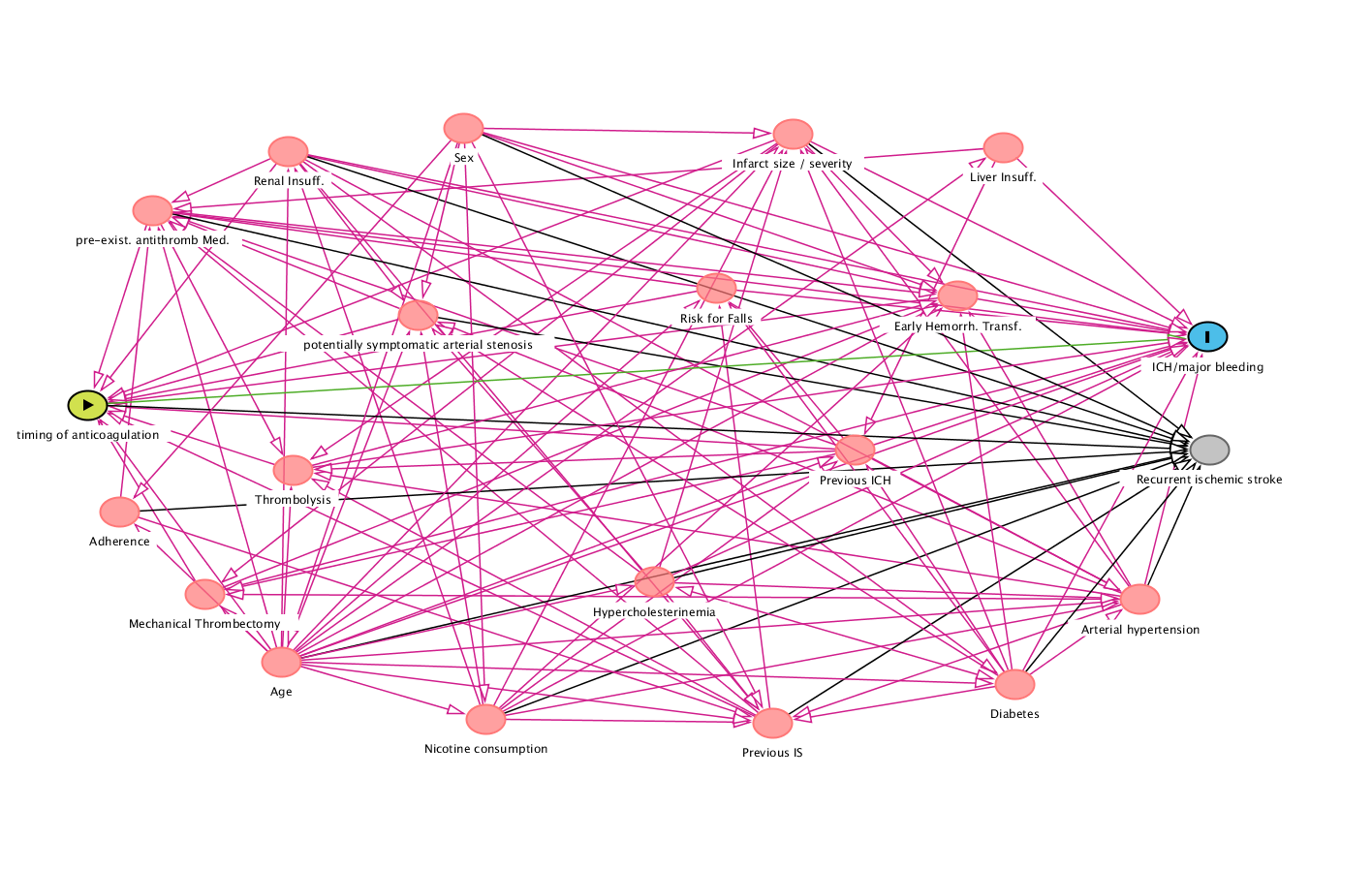


**PRODAST Investigators^21^**

| Darius G. Nabavi | Klinik für Neurologie mit Stroke Unit, Klinikum Neukölln, Vivantes Netzwerk für Gesundheit GmbH, Berlin |
| --- | --- |
| Paul Sparenberg | Klinik für Neurologie mit Stroke Unit und neurologischer Frührehabilitation, BG Klinikum Unfallkrankenhaus Berlin, Berlin |
| Karin Weissenborn,  Gerrit M. Grosse | Klinik für Neurologie, Medizinische Hochschule Hannover, Hannover |
| Klaus Gröschel, Timo Uphaus | Klinik und Poliklinik für Neurologie, Universitätsmedizin Mainz, Mainz |
| Dominik Michalski | Department of Neurology, University of Leipzig, Leipzig |
| Sven Poli | Department of Neurology & Stroke, Eberhard-Karls University, Tübingen, Germany, Hertie Institute for Clinical Brain Research, Eberhard-Karls University, Tübingen |
| Georg Royl,  Susanne Riebau | Department of Neurology and Center of Brain, Behavior and Metabolism , University of Lübeck, Lübeck |
| Waltraud Pfeilschifter,  Jan Hendrik Schaefer | Department of Neurology, Goethe University, Frankfurt am Main |
| Hassan Soda, Renate Weinhardt | Klinik für Akutneurologie/Stroke Unit und Intensivmedizin, RHÖN-KLINIKUM Campus Bad Neustadt, Bad Neustadt |
| Ruediger Hilker-Roggendorf | Department of Neurology, Klinikum Vest, Recklinghausen |
| Carsten Pohlmann | Abteilung für Neurologie, Asklepios Klinik Barmbek, Hamburg |
| Peter Kraft | Neurologische Abteilung, Klinikum Main-Spessart, Lohr |
| Nikola Popovic | Klinik für Neurologie, Evangelisches Krankenhaus Hattingen, Hattingen |
| Frank Hamilton,  Bruno-Marcel Mackert | Vivantesklinikum Auguste-Viktoria, Klinik für Neurologie mit Stroke Unit, Berlin |
| Arya Bagherzadeh-Khorsandi | Klinik für Neurologie und Neurogeriatrie, Kreisklinikum Siegen, Siegen |
| Jens D. Rollnik | BDH-Klinik Hessisch Oldendorf gGmbH, Institut für neurorehabilitative Forschung (InFo), Assoziiertes Institut der Medizinischen Hochschule Hannover (MHH), Hessisch Oldendorf |
| Lars Marquardt | University Department of Neurology, Asklepios Hospital Hamburg Wandsbek, Germany, Faculty of Medicine, Semmelweis University Campus Hamburg, Hamburg |
| Peter Kühnlein | Neurologische Klinik, Klinikum Coburg GmbH, Coburg |
| Andrea Kraft | Department of Neurology, Martha-Maria Hospital Halle, Halle |
| Götz Thomalla | Klinik und Poliklinik für Neurologie, Kopf- und Neurozentrum, Universitätsklinikum Hamburg-Eppendorf, Hamburg |
| Bernhard Wallner | Abteilung für Neurologie, Knappschaftskrankenhaus Bottrop, Bottrop |
| Peter D. Schellinger | Dept. of Neurology and Neurogeriatrics, JW Medical Center Minden, UK RUB, Minden |
| Gabor Petzold | Neurologische Klinik, Universitätsklinikum Bonn, Bonn |
| Bettina Schmitz | Klinik für Neurologie, Stroke Unit und Zentrum für Epilepsie, Neurologische Früh-Rehabilitation, Vivantes Humboldt-Klinikum Berlin, Berlin |
| Marcus Thieme | Fachbereich Innere, REGIOMED Klinikum Sonneberg, Sonneberg |
| Lars Krause | Klinik für Neurologie und Neurologische Frührehabilitation, Klinikum Osnabrück, Osnabrück |
| Peter A. Ringleb | Neurologische Universitätsklinik Heidelberg, Heidelberg |
| Johann Lambeck | Department of Neurology and Neurophysiology, University Medical Center Freiburg, Freiburg |
| Jörg Berthel | Klinik für Neurologie, Klinikum Fulda, Universitätsmedizin Marburg-Campus Fulda, Fulda |
| Felix Butscheid | Neurologie, Krankenhaus Buchholz , Buchholz |
| Andreas Binder,  Johannes Meyne | Klinik für Neurologie, Universitätsklinikum Schleswig-Holstein, Campus Kiel, Christian-Albrechts-Universität zu Kiel, Kiel |
| Klaus Jahn | Schoen Clinic Bad Aibling, Bad Aibling |
| Arne Jacobs | Department of Neurology, Sanaklinik Lübeck, Lübeck |
| Christoffer Kraemer | Klinik für Neurologie und Klinische Neurophysiologie, Klinikum Lüneburg, Lüneburg |
| Ralph Weber | Klinik für Neurologie, Alfried Krupp Krankenhaus Essen, Essen |
| Peter Wienecke | Neurologie, Asklepios Fachklinikum Teupitz, Teupitz |
| Marc E. Wolf | Neurologische Klinik, Klinikum Stuttgart, Stuttgart |
| Roger Schubert | Fachabteilung Neurologie, SRH Wald-Klinikum Gera GmbH, Gera |
| Wolfgang Heide | Neurologische Klinik, AKH Celle, Celle |
| Heiko Dietzel | Abteilung für Neurologie, Krankenhaus St. Elisabeth Damme, Damme |
| Michael Görtler | Klinik für Neurologie, Universitätsklinikum Magdeburg, Magdeburg |
| Christos Krogias | Dept. of Neurology, St. Josef-Hospital, Ruhr University Bochum, Bochum |
| Jessica Barlinn | Department of Neurology, University Hospital Carl Gustav Carus, Technische Universität Dresden, Dresden |
| Sabine Mehnert | Department of Neurology, Sana Hanse Hospital Wismar, Wismar |
| Pawel Kermer | Department of Neurology, Nordwest-Krankenhaus Sanderbusch gGmbH, Sande |
| Götz Greif | Fachklinik Neurologie, Kliniken Maria Hilf Mönchengladbach, Mönchengladbach |
| Florian Bethke | Klinik für Neurologie, Klinikum Ibbenbüren, Ibbenbüren |
| Ulrich Pulkowski | Neurologie mit Stroke Unit, imland Klinik Rendsburg, Rendsburg |
| Gernot Reimann | Stroke-Unit und Neurologische Intensivstation, Klinikum Dortmund, Dortmund |
| Christiane Weck | Neurologie, Krankenhaus Agatharied, Hausham |
| Jan Liman | Klinik für Neurologie, Universitätsmedizin Göttingen, Göttingen |
| Karl Georg Haeusler | Neurologische Klinik und Poliklinik, Universitätsklinikum Würzburg, Würzburg |
| Holger Grehl | Neurologische Klinik, Ev. Klinikum Niederrhein, Krankenhaus Duisburg-Fahrn, Duisburg |
| Daniel Ostertag | Klinik für Neurologie, Stroke Unit, Früh-Reha & Schmerzklinik, Klinikum Merzig, Merzig |
| Jens Minnerup | Klinik für Neurologie mit Institut für Translationale Neurologie, Universitätsklinikum Münster, Münster |
| Michael Bauerle | Klinik für Neurologie, Klinikum Emden, Emden |
| Patrick Oschmann | Department of Neurology, Klinikum Bayreuth GmbH, Bayreuth |
| Björn Wito Walther | Fachabteilung Neurologie, SRH Zentralklinikum Suhl GmbH, Suhl |
| Franz Blaes | Fachabteilung Neurologie, Klinikum Oberberg, Kreiskrankenhaus Gummersbach, Gummersbach |
| Hakan Cangür | Department of Neurology & Stroke, Wolfsburg Hospital, Wolfsburg |
| Muhterem Erinola | Neurologische Klinik, St. Marien Hospital Lünen, Lünen |
| Andreas Dietz | Klinik für Neurologie, Hochtaunus-Kliniken gGmbH, Krankenhaus Bad Homburg, Bad Homburg |
| Birgit Herting | Klinik für Neurologie und Gerontoneurologie, DIAKONEO Diak Klinikum, Diakonie-Klinikum Schwäbisch Hall gGmbH, Schwäbisch Hall |
| Torsten Ruck | Neurologie, SHR Klinikum Karlsbad-Langensteinbach, Karlsbad |
| Christian Urbanek | Neurologische Klinik, Klinikum der Stadt Ludwigshafen am Rhein, Ludwigshafen |
| Jochen Machetanz | Neurologische Klinik Friedrichstadt, Städtisches Klinikum Dresden, Dresden |
| Christian Oelschläger | Klinik für Neurologie, Evangelisches Klinikum Bethel, Bielefeld |
| Susanne Müller | Department of Neurology, University of Ulm, Ulm |
| Sara Friederike Heunecke | Abteilung Neurologie, Asklepios Klinik Schildautal Seesen, Seesen |
| Sylke Düllberg-Boden | Fachabteilung Neurologie, Evangelisches Krankenhaus Herne, Herne |
| Torsten Rehfeldt | Klinik für Neurologie, Dietrich-Bonhoeffer-Klinikum Neubrandenburg, Neubrandenburg |
| Olav Schwarte | Klinik für Neurologie, InnKlinikum Altötting und Mühldorf, Altötting |
| Anne Kesseler | Klinik für Neurologie, Rhein-Maas Klinikum Würselen, Würselen |
| Jens-Holger Moll | Klinik für Neurologie, Krankenhaus Maria-Hilf Krefeld, Krefeld |
| Tobias Müller,  Bernhard Sehm | Department of Neurology, University of Halle-Wittenberg, Halle (Saale), Sachsen-Anhalt, Halle (Saale) |
| Jörg Berrouschot | Klinik für Neurologie, Klinikum Altenburger Land GmbH, Altenburg |
| Jens Diekmann | Klinik für Neurologie und klinische Neurophysiologie, DIAKOVERE Henriettenstift, Hannover |
| Jana Zarzitzky | Klinik für Neurologie, Klinikum Frankfurt Höchst GmbH, Kliniken Frankfurt-Main-Taunus GmbH, Frankfurt am Main |
| Jan Wehnemann | Klinik für Neurologie, Ammerland Klinik, Westerstede |
| Matthias von Mering | Klinik für Neurologie, Klinikum Bremen-Nord, Bremen |
| Lukas Kremmler | Klinik für Neurologie, Krankenhaus Barmherzige Brüder Regensburg, Regensburg |
| Ludwig Niehaus | Neurologie, Rems-Murr-Klinikum Winnenden, Winnenden |
